# Development of a sandwich ELISA to detect *Leishmania* 40S ribosomal protein S12 antigen from blood samples of visceral leishmaniasis patients

**DOI:** 10.1186/s12879-018-3420-2

**Published:** 2018-10-03

**Authors:** Wen-Wei Zhang, Ayan Kumar Ghosh, Raodoh Mohamath, Jacqueline Whittle, Alessandro Picone, Patrick Lypaczewski, Momar Ndao, Randall F Howard, Pradeep Das, Steven G Reed, Greg Matlashewski

**Affiliations:** 1 Department of Microbiology and Immunology, McGill University, 3775 University Street, Montreal, Quebec, H3A2B4 Canada; 2 Rajendra Memorial Research Institute of Medical Sciences (ICMR), Patna, Bihar India; 3 Infectious Disease Research Institute, Seattle, WA USA; 4 National Reference Center for Parasitology, The Research Institute of the McGill University Health Center, Montreal, Quebec, Canada

**Keywords:** Visceral Leishmaniasis, Diagnosis, ELISA, PBMC, 40S ribosomal protein S12

## Abstract

**Background:**

Visceral leishmaniasis (VL), caused by *Leishmania donovani* complex parasites, is a neglected parasitic disease that is generally fatal if untreated. Despite decades of research to develop a sensitive VL diagnostic test, definitive diagnosis of VL still mainly relies on the visualization of the parasite in aspirates from the spleen, liver or bone marrow, an invasive and dangerous process with variable sensitivity. A sensitive assay that can detect *Leishmania* antigen from blood samples will help confirm cause, cure or recurrence of VL.

**Methods:**

In this study, rabbit polyclonal antibodies were raised against eight recombinant *Leishmania* proteins that are highly abundant in *Leishmania*. The antibodies were purified and labeled with biotin for developing a prototype sandwich enzyme-linked immunosorbent assay (ELISA).

**Results:**

The ELISA for the *Leishmania* 40S ribosomal protein S12 detected target antigen with the highest sensitivity and specificity and could detect 1 pg of purified protein or as few as 60 *L. donovani* parasites. The 40S ribosomal protein S12 sandwich ELISA could detect the target antigen from Peripheral Blood Mononuclear Cell (PBMC) samples in 68% of VL patients and post-kala-azar dermal leishmaniasis (PKDL) patients, providing an estimation of parasitemia ranging from 15 to 80 amastigotes per ml of blood.

**Conclusion:**

These results indicate that the 40S ribosomal protein S12 sandwich ELISA warrants further tests with more clinical samples of VL patients and other parasitic diseases. It is hopeful that this ELISA could become a useful tool for confirming VL diagnosis, monitoring treatment progress, disease recurrence and possibly detecting asymptomatic *Leishmania* infections with a high parasite load.

## Background

Leishmaniasis is caused by the protozoan *Leishmania* parasites, which are transmitted by the bite of infected sandflies. Depending on the infecting species, *Leishmania* infection can cause cutaneous leishmaniasis (CL), mucocutaneous leishmaniasis (MCL) or visceral leishmaniasis (VL). VL, also known as kala-azar, is the most serious form of the disease and is frequently fatal if left untreated [1, 2]. VL is highly endemic in the Indian subcontinent, East Africa and parts of South America. An estimated 50,000 to 90,000 new cases of VL occur worldwide each year (http://www.who.int/news-room/fact-sheets/detail/leishmaniasis). Due to the AIDS epidemic, coinfection with human immunodeficiency virus (HIV) has increased VL cases in some parts of the world [3]. In addition, *Leishmania infantum* infection causes visceral disease in domestic dogs, which are the major vertebrate reservoirs for transmission to humans in Latin America and Southern Europe [4, 5].

VL is characterized by irregular bouts of fever, weight loss, enlargement of the spleen and liver, and anemia. However, these clinical features are not specific and can be mistaken for other common illnesses associated with fever including malaria. Moreover, infection with *Leishmania* does not always lead to clinical illness, asymptomatic infections are common, and it is unknown whether these individuals represent a source of transmission [6, 7]. Although there are some drawbacks associated with the current treatments, VL is a life-threatening disease that is curable with proper treatment [7]. Therefore, rapid and accurate diagnosis of visceral *Leishmania* infection is important for patients to receive prompt treatment, determine cure or an indication of relapse, and thus prevent further transmission of this disease [7].

Currently, diagnosis of VL is made by combining clinical symptoms with parasitological or serological tests. Assays based on detection of parasite-specific antibodies (such as the rK39 test) have proven to be efficient for VL diagnosis. The rK39 immunochromatographic test (ICT) is easy to perform, rapid and inexpensive. However, because the rK39 ICT detects antibodies, it cannot distinguish relapse cases from past infection, or active disease from asymptomatic infection and cannot be used as a test of cure [8–10]. The rK39 ICT is less effective in VL patients co-infected with HIV and is more sensitive for VL diagnosis in Asia than in Africa [8–10], though the new rK28 ICT has improved the detecting sensitivity of VL cases in Africa [11].

Nucleic acid-based diagnostics such as polymerase chain reaction (PCR) are the most sensitive method to detect the presence of parasites in clinical samples, but they are expensive and restricted to referral hospitals and research centers, though this situation could be improved with development of loop-mediated isothermal amplification (LAMP) assays where there has been recent progress [12–17]. Definitive diagnosis of VL still requires microscopic identification of the parasite in internal organs such as in spleen, liver or bone marrow aspirates, an invasive and dangerous process with varied sensitivity (53–99%) [8–10]. Therefore, development of an assay that can sensitively detect *Leishmania* antigen from blood or urine samples would be helpful for rapid and definitive VL diagnosis, test of cure and relapse [18–24].

Based on the hypothesis that abundant *Leishmania* proteins could be easier to detect than low abundance proteins, we raised rabbit polyclonal antibodies against eight *Leishmania* proteins previously reported to be highly abundant in *Leishmania* [25–27]*.* With these rabbit antisera, we developed a direct enzyme-linked immunosorbent assay (ELISA), and a sandwich ELISA with purified antibodies labeled with biotin for detection of the *Leishmania* antigens. The sandwich ELISA against the *Leishmania* 40S ribosomal protein S12 provided the highest sensitivity and specificity. Importantly, the sandwich ELISA could detect *Leishmania* 40S ribosomal protein S12 antigen in PBMC lysates prepared from VL patients and post-kala-azar dermal leishmaniasis (PKDL) patients. These results suggest that the 40S ribosomal protein S12 sandwich ELISA could represent a useful test for confirming VL diagnosis and for monitoring treatment progress and relapse.

## Methods

### Selection of abundant *Leishmania* proteins

The ten soluble and abundant *Leishmania* proteins we selected (Table 1) were based on previous *Leishmania* proteomic analysis, these proteins were present as large and dense spots in two-dimension gel electrophoresis and were identified using mass spectrometry [25, 26]. The abundance of these selected proteins was also confirmed by our own more recent proteomic study on *L. donovani* [27]. Most of these abundant *Leishmania* proteins except aldolase (LmjF.36.1260) have low homology to human proteins.

**Table 1:** A list of abundant *Leishmania* proteins selected for production of recombinant proteins in *E. coli*

Systematic Name	Simplified Name	Encoded Protein	Molecular weight (kDa)
LmjF.05.0350	535^a^	Trypanothione reductase	53.1
LmjF.05.0450	545	Kinetochore related protein	22.3
LmjF.05.0830	583	Methylthioadenosine phosphorylase	33.4
LmjF.13.0570	1357	40S ribosomal protein S12	15.6
LmjF.24.1500	2415	Translationally controlled tumor protein	19.4
LmjF.32.0460	3246^a^	Prostaglandin F synthase	31.8
LmjF.35.0820	3582	Aspartate aminotransferase	45.9
LmjF.24.2110	24,211	3-Hydroxy-3-methyl glutaryl-CoA synthase	55.2
LmjF.36.1260	36,126	Fructose-1,6-bisphosphate aldolase	40.8
LmjF.36.6760	36,676	ATP synthase delta (OSCP) subunit	28.9

^a^Because of low yield, these proteins were not sent for production of antisera

### Construction of bacterial expression vectors

For convenience of cloning, we have replaced the sequence (*Nde* I to *Not* I) containing the S. Tag and the multiple cloning sites in pET29 bacterial expression vector (Novagen) with following sequence containing a His-Tag sequence and new multiple cloning sites (*Hind* III, *Kpn* I, *EcoR* I, *BamH* I, *Bgl* II and *Not* I): CATATGGCACATCACCACCACCATCACAAGCTTGGTACCGAATTCGGATCCAGATCT GTAGCGGCCGC. We re-named the modified pET29 vector as pET29w. Accordingly, the primers with corresponding restriction enzyme site added at the 5′ end were designed and used to amplify the gene sequences of these abundant *Leishmania* proteins by PCR using *L. donovani* genomic DNA as the template. The *Leishmania* gene PCR products with expected sizes were digested with restriction enzymes and cloned into the corresponding sites of pET29w bacterial expression vector. A list of these primer pairs is shown in Table 2.

**Table 2:** A list of primers used to amplify genes encoding abundant *Leishmania* proteins

Systematic Name	Primer pairs used for PCR	Gene size (bp)
LmjF.05.0350	5’ cccaagcttaccATGTCCCGCGCGTACGACCTCG	1476
	5’ ggaagatctgtGAGGTTGCTGCTCAGCTTTTCG	
LmjF.05.0450	5’ cccaagcttaccATGGCTGACGAAGGCGCTATAGA	591
	5’ ggaagatctgtCTTCTTCGTCGTGGCCTTCACAG	
LmjF.05.0830	5’ cccaagcttaccATGTACGGCAACCCGCACAAGGA	921
	5’ ggaagatctgtCGGGGCGAACTGCGGGTACTTGC	
LmjF.13.0570	5’ cggggtaccATGGCTGAGGAAACCGTCCGTGTTG	426
	5’ cgcggatccgtGTGCAGCTGAGACAGCAGGTAGTCC	
LmjF.24.1500	5’ cggggtaccATGAAGATCTTCAAGGACGTGCTG	513
	5’ cgcggatccgtGACGCGCTCGCCCTTCAAGCCATC	
LmjF.32.0460	5’ cccaagcttaccATGGCTGTTAAGTGCACGCACG	840
	5’ ggaagatctgtCTTGCGCTCGGTTGGGAAGAAGG	
LmjF.35.0820	5’ cccaagcttaccATGTCCACGCAGGCGGCCATG	1239
	5’ ggaagatctgtCTCACGATTCACATTGCGCACA	
LmjF.24.2110	5’ cggggtaccATGATGCGCAACACCTGTCTTAG	1506
	5’ cgcggatccgtCTGGATGTAGCGGTAGTACTCA	
LmjF.36.1260	5’ cccaagcttaccATGTCGCGTGTGACGATCTTTCAG	1116
	5’ cgcggatccgtATAGATGTTGCCTTTGACGTACAG	
LmjF.36.6760	5’ cggggtaccATGTTCCGCCGTCTCTCCGTG	777
	5’ cgcggatccgtAACACCGCTCTTGAGCTCCTCG	

### Expression and purification of recombinant *Leishmania* proteins in *E. coli* BL21 or Rosetta cells

We isolated the recombinant *Leishmania* proteins from bacteria by following the manufacturer’s protocol (Novagen pET system manual). Some of these recombinant proteins formed Inclusion Bodies after Isopropyl β-D-1-thiogalactopyranoside (IPTG) induction of the culture and were first urea-solubilized before using Ni-NTA Agarose resin (Qiagen) to purify. Depending on the protein, we obtained 2–10 mg of each recombinant protein from 1 L shake cultures for polyclonal antibody production.

### Production of rabbit polyclonal antibodies

The rabbit polyclonal antibodies (antisera) against these abundant *Leishmania* proteins were produced by Syd Labs, Inc. (Natick, MA, USA). The detailed procedures can be found in the website of Syd Labs, Inc. (https://www.sydlabs.com). One or two rabbits were immunized for each *Leishmania* recombinant protein with Freund’s adjuvant (SIGMA, USA). About 0.5 ml of pre-immune rabbit serum and 40 to 80 ml of antiserum with ELISA titers > 1:20,000 were obtained within 4 months for each antigen. The antisera were kept at 4 °C for current use and in a − 20 °C freezer for longer storage.

### *Leishmania* strains and macrophage infection

The *L. donovani* 1S/Cl2D and *Leishmania major* Friedlin V9 strains used in this study were routinely cultured at 27 °C in M199 medium (Cat # M0393, SIGMA) supplemented with 10% heat-inactivated fetal bovine serum, 40 mM HEPES (pH 7.4), 0.1 mM adenine, 5 mg l^− 1^hemin, 1 mg l^− 1^ biotin, 1 mg l^− 1^ biopterine, 50 U ml^− 1^ penicillin and 50 μg ml^− 1^ streptomycin. Cultures were passaged to fresh medium in a 20-fold dilution once a week. Infection of cultured macrophages was performed as described [28].

### Western blot analysis of abundant *Leishmania* proteins

Whole cell lysates were prepared in sodium dodecyl sulfate polyacrylamide gel electrophoresis (SDS-PAGE) sample buffer from *L. major* promastigotes, *L. donovani* promastigotes, *L. donovani* axenic amastigotes, and human H1299 epithelial cells. After boiling in water for 3 min and centrifuging at 15,000 RPM (Revolutions per minute) for 10 min, the cell lysate supernatants were separated in SDS-PAGE gel and subjected to Western blot analysis [29]. Rabbit antiserum was diluted 1:1000 for Western blot analysis to detect target proteins in *Leishmania* cell lysates (4 × 10^7^ *Leishmania* cells per lane).

### Isolation of peripheral blood mononuclear cells (PBMCs) from clinical samples

The diagnosis for most of the VL patients at the Rejandra Memorial Research Institute of Medical Sciences (RMRIMS), Patna, Bihar was confirmed by spleen or bone marrow biopsies. PKDL cases at RMRIMS were confirmed by skin smear microscopy for LD bodies (Amastigotes). The healthy controls were all rK39-negative from the out-patient department for regular check-up. Total 14 VL patients, 5 likely VL cases which had typical VL symptoms and were rK39-positive but biopsy-negative, 3 PKDL patients and 12 healthy controls were recruited. The 14 VL cases included 10 males with age range from 3 to 52 years old and 4 females with age range 18 to 35; the 5 likely VL cases were 3 males with age range 14 to 45 and 2 females with age 40 and 45; the 3 PKDL patients included 1 male aged 30, and 2 females aged 18 and 40; the 12 healthy controls were 7 males and 5 females with age range from 24 to 34. Blood (5 ml per patient) drawn from patients before treatment were utilized for preparation of PBMCs with Ficoll-density separation [30]. PBMC lysates were prepared by lysing cells in 1% NP-40 in PBS with proteinase inhibitors (cOmplete Cocktail Tablets, SIGMA) at the concentration of 1 × 10^7^ cells/ml. After incubation on ice for 30 min with occasional mixing, the lysates were centrifuged at 4 °C at high speed (15,000 RPM) for 15 min, and the supernatant was kept and stored at -20 °C until use.

### Direct ELISA

*L. donovani* promastigote lysate was prepared by lysing cells in 1% NP-40 in PBS (Phosphate-buffered saline) with proteinase inhibitors at the concentration of 4 × 10^8^ cells/ml. After centrifugation at 4 °C at high speed for 15 min, the supernatant was stored at -20 °C until use.

The ELISA plate (Immulon cat#: 62402–972 from VWR) was coated with 50 μl (per well) cell lysate diluted in carbonate/bicarbonate buffer (1.89 g NaHCO_3_ and 0.954 g Na_2_CO_3_ in 500 ml H_2_O, pH 9.6) and incubated at 4 °C in a moist chamber overnight. The plate was washed 2 times with 200 μl wash buffer (0.05% Tween 20 in PBS) then blocked with 200 μl/well blocking buffer (5% nonfat dry milk in 0.1% PBS/T) for 1 h at 37 °C (covered with an adhesive plastic). The plate was washed 2 times with wash buffer, and 100 μl rabbit antiserum in 1:2000 dilution in blocking buffer was added to each well for 1.5 h at 37 °C or overnight at 4 °C. The plate was washed 3 times with wash buffer and 100 μl of horseradish peroxidase (HRP)-linked anti-rabbit antibody (ECL NA934V 1:5000 in blocking buffer) was added into each well for 1 h at 37 °C. The plate was washed 4 times with wash buffer followed by adding 50 μl/well of the HRP substrate TMB (eBioscience). After 10 min incubation at room temperature, the reactions were stopped with 25 μl of 1 N H_2_SO_4_. The color change in wells was read at 450 nm absorbance.

### Purification and labelling of rabbit anti-*Leishmania* protein antibodies

To set up the sandwich ELISA, Immunoglobulin G (IgG) was first purified from the rabbit antisera with the Melon Gel IgG Purification System (Cat # 45206, Thermo Fisher Scientific), which purifies antibodies from serum by removing non-relevant proteins. After dilution with the Melon gel purification buffer (1:10) and passing through the Melon gel purification column, 100 μl of serum generated 1000 μl of purified IgG. One-half of the purified IgGs were biotin labeled using the Thermo Scientific EZ-Link Sulfo-NHS-SS-Biotin (sulfosuccinimidyl-2-[biotinamido]ethyl-1,3-dithiopropionate, Cat # 21331). 7 μl Sulfo-NHS-SS-Biotin (6 mg/ml, freshly prepared) was added into 500 μl purified rabbit IgG and labeled at 4^o^ C overnight. Free Sulfo-NHS-SS-Biotin was removed from the labeling reaction by dialysis in PBS.

### Sandwich ELISA

A 96-well ELISA plate was coated with 100 μl/well of capture antibody in carbonate/bicarbonate coating buffer (i.e. 0.5 μl capture ab + 100 μl coating buffer; 1:200 dilution). The plate was sealed and incubated overnight at 4 °C in a moist chamber. Wells were aspirated and washed 5 times with 250 μl/well wash buffer (0.05%Tween 20 in PBS) allowing time for soaking (~ 1 min) during each wash step. Absorbent paper was used to remove any residual buffer. Wells were blocked with 200 μl/well of 2% bovine serum albumin (BSA) in PBS, incubated at 37 °C for 2 h, and aspirated and washed 2 times. A 2-fold serial dilution of the standards (*L. donovani* promastigote lysate alone or mixed with the healthy control PBMC lysate) was performed with assay buffer (1% BSA in wash buffer) with a final volume of 100 μl per well to generate the standard curve. PBMC lysates of clinical samples (100-250 μl) prepared as above were added into each well. The plate was covered, incubated overnight at 4 °C, aspirated free of buffer, and washed 5 times. Detection antibody (100 μl/well) diluted in assay buffer (i.e., 0.25 μl Detection ab-Biotin + 100 μl Assay buffer; 1:400 dilution) was added per well, and the plate was sealed and incubated at 37 °C for 2 h. Wells were aspirated and washed 5 times, and 100 μl/well of Avidin-HRP (eBioscience) diluted in assay buffer (1:250 dilution) was added and incubated at room temperature for 30 min to 1 h. Wells were aspirated and washed 10 to 14 times with the wells soaking for 2 min for each wash. Substrate solution (TMB; 100 μl/well) was added, and plates incubated at room temperature for 15 min. The reactions were stopped by adding 50 μl/well of Stop Solution (2 N H_2_SO_4_), and absorbance was read at 450 nm.

### Generation of monoclonal antibodies against peptides of *L. donovani* 40S ribosomal protein S12

Monoclonal antibodies against *L. donovani* 40S ribosomal protein S12 peptides were generated by Abmart (Shanghai) Co. Ltd. However, none of these anti-peptide monoclonal antibodies could recognize the native *L. donovani* 40S ribosomal protein S12 (See discussion). The twelve peptide sequences used to generate the monoclonal antibodies are as follows: NVVVDVAPES,EETVRVEVPA,EVPAVEENVV,VAGEVTKTLK,QKALEANGLV,FGERTKALDY,ESLEDAVRIV,NVEEREKLAQ,TALAKQGNID,VRGLSEVART,QWAGLVRRDV,EDEEYKKLVT.

## Results

### Expression and purification of recombinant *Leishmania* proteins in *E. coli*

**Fig. 1:**
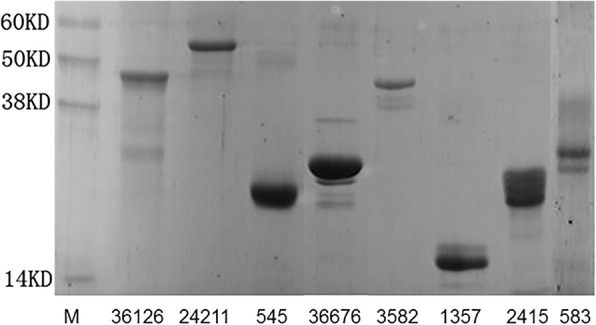
Production and purification of the recombinant abundant *Leishmania* proteins. The eight abundant *Leishmania* protein genes were PCR amplified from *L. donovani* and cloned into the pET29w expression vector. Recombinant proteins containing an N-terminal His-tag were expressed in *E. coli* BL21 and purified with Ni-NTA agarose. Purity was evaluated by SDS-PAGE and Coomassie blue staining. These recombinant *Leishmania* proteins were sent out for rabbit polyclonal antibody production

We hypothesized that abundant *Leishmania* proteins would be more easily detected from clinical samples than low abundance proteins. Therefore, we selected ten soluble and abundant *Leishmania* proteins that were previously identified by proteomic analysis using two-dimension gel electrophoresis and mass spectrometry [25, 26] for production as recombinant proteins in *E. coli* (Table 1). The coding sequences of these abundant *Leishmania* proteins were amplified by PCR from *L. donovani* genomic DNA and cloned into the pET29w expression vector, placing the His-tag at the N-terminus of the expressed recombinant proteins. The His-tagged proteins were purified from *E. coli* lysates on nickel-charged resin columns and the purity was verified by SDS-PAGE (Fig. 1). Eight of the recombinant proteins that met the required yield and purity were used to immunize rabbits to produce polyclonal antisera.

### Western blot analysis of antisera against *Leishmania* and human cells

**Fig. 2:**
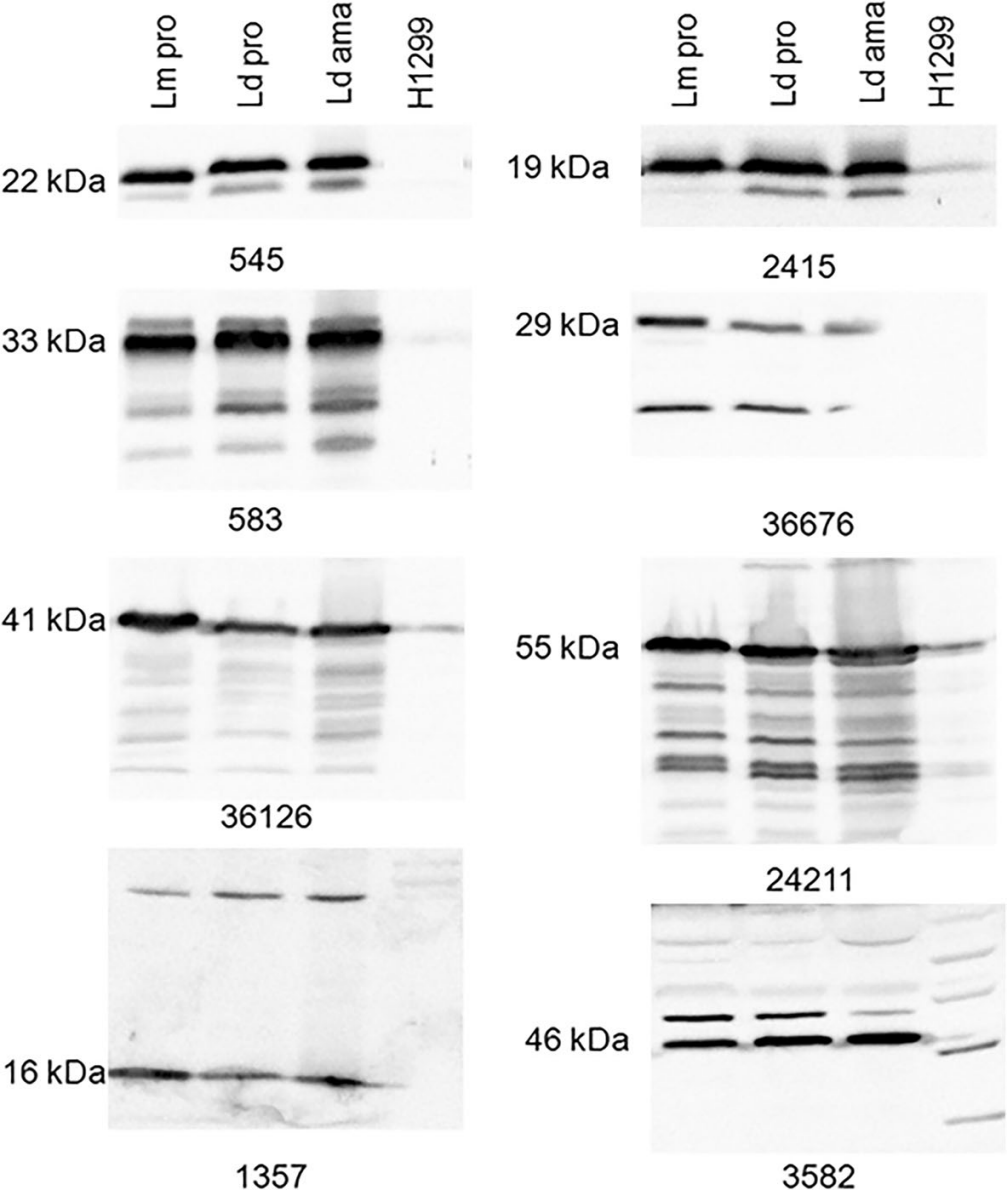
Western blot analysis show all eight rabbit antisera generated in this study specifically detected the corresponding *Leishmania* proteins. The lysate of 4 × 10^7^ *Leishmania* cells or 1.5 × 10^5^ Human H1299 cells was loaded into each lane and separated on SDS-PAGE. The specific *Leishmania* proteins were detected with rabbit antisera in 1:1000 dilution and goat anti-rabbit IgG labeled with horseradish peroxidase. Lm pro: *L. major* promastigotes; Ld pro: *L. donovani* promastigotes; Ld ama: *L. donovani* axenic amastigotes; H1299: Human H1299 cell line. Note: similar amounts of these abundant *Leishmania* proteins were detected in *L. major*, *L. donovani* promastigotes, and *L. donovani* axenic amastigotes, while these sera exhibited variable cross reactivities to human proteins in the H1299 cell lysate

To determine whether the resulting antisera could specifically recognize the *Leishmania* proteins, whole cell lysates prepared from *L. major* promastigotes, *L. donovani* promastigotes, *L. donovani* axenic amastigotes, and human H1299 epithelial cells were separated in SDS-PAGE gel and subjected to Western blot analysis. As shown in Fig. 2, all the rabbit antisera could detect the corresponding *Leishmania* proteins of the correct size. Importantly, *Leishmania* proteins were equally well expressed in *L. major* promastigotes, *L. donovani* promastigotes, and *L. donovani* axenic amastigotes, though some had one or multiple detectable non-specific bands depending on the antisera. These additional protein bands could be the partially degraded protein products (smaller bands for 545, 2415 and 36,676), products with post translation modifications or oligomers (larger bands for 1357 and 3582 proteins), or cross reaction with other proteins (the multiple additional bands seen for 583, 36,126 and 24,211). Except for anti-3582 serum, these antisera had no or low cross reactivity to human proteins in H1299 cell lysate.

### ELISA analysis of antisera against *L. donovani* proteins

**Fig. 3:**
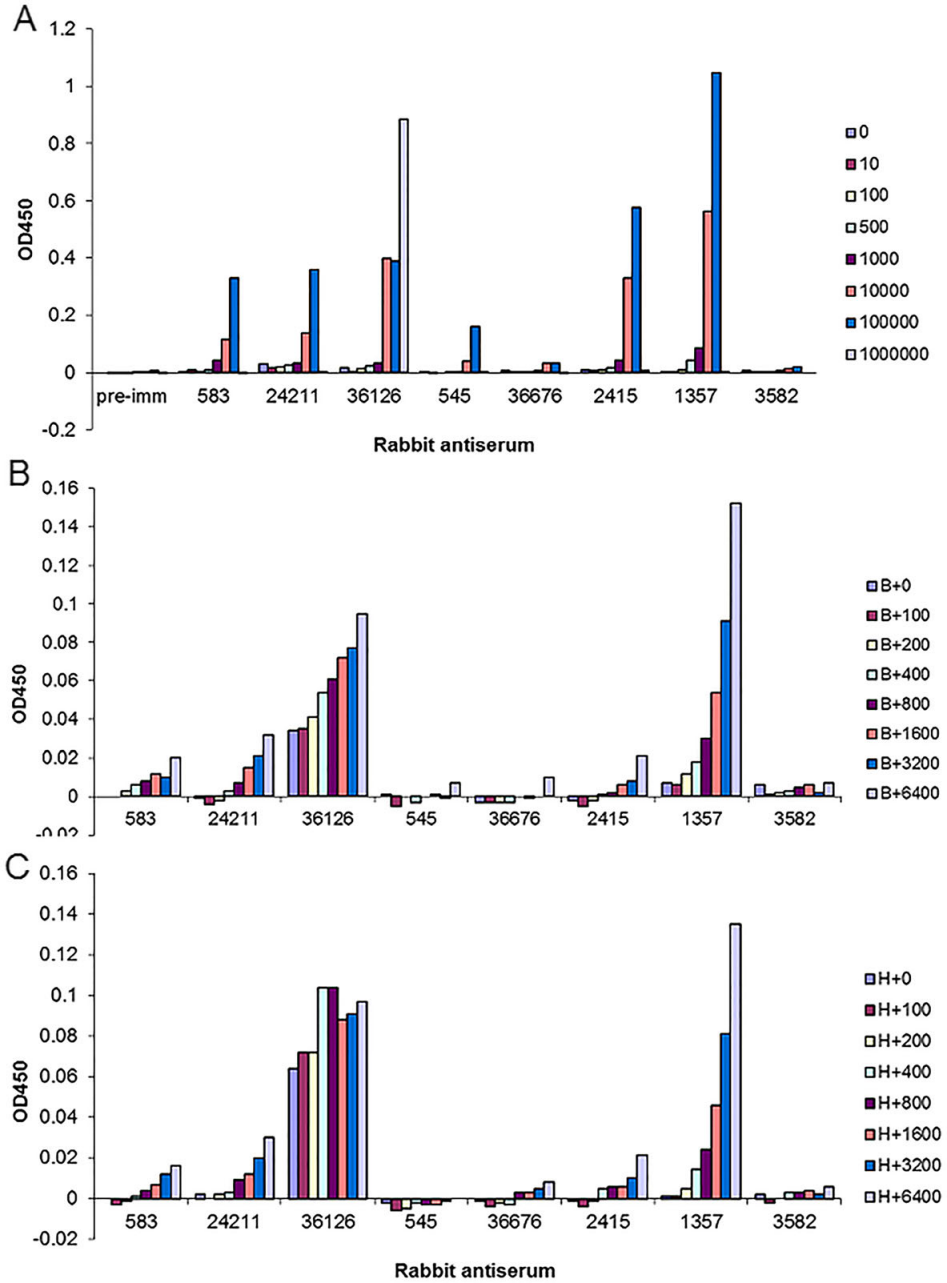
The rabbit serum against *Leishmania* 40S ribosomal protein S12 antigen (1357) displayed the highest sensitivity and specificity in a direct ELISA. To determine whether these rabbit antisera can detect the corresponding antigen in *L. donovani* cell lysate in traditional ELISA, ELISA plates were directly coated with *L. donovani* promastigote lysate ranging from 10 to 1 million promastigotes per well as indicated in **a**. Each rabbit antiserum in 1:2000 dilution was added to the plate, followed by adding the HRP-linked anti-rabbit antibody and HRP substrate TMB. The color change proportional to the level of target antigen detected was measured at A450nm. **b** and **c** To determine whether these antisera are able to detect *Leishmania* antigen in *L. donovani*-infected macrophages, the lysate of *L. donovani*-infected B10R-mouse macrophages were mixed with non-infected B10R macrophage lysate (**b**) or human H1299 cell lysate (**c**) in different ratios (denoted as B or H in the figure legends, respectively) and coated on the plate so that each well contained the same amount of cell lysate from a total 3000 (infected plus non-infected) mammalian cells where the total number of amastigotes ranged from 100 to 6400 per well, as indicated; the plate was then hybridized with these rabbit antisera as **a**

To establish the sensitivity of these rabbit antisera in detecting *L. donovani* antigens in an ELISA format, microtiter plates were coated with *L. donovani* promastigote lysates (ranging from 10 to 1 million promastigotes per well). Rabbit antisera were diluted 1: 2000 and added to the plate followed by the HRP-linked anti-rabbit antibody and the HRP substrate TMB, and the color change at 450 nm absorbance were measured. As shown in Fig. 3a, six of the eight antisera could detect the corresponding antigen from the *L. donovani* cell lysate in this assay (36,676 and 3582 were the exceptions). Antiserum for 1357 (40S ribosomal protein S12) showed the highest sensitivity with a detection limit of approximately 500 *L. donovani* promastigotes per well.

To determine whether these antisera could detect *Leishmania* antigen in *L. donovani*-infected macrophages, mouse macrophage B10R cells were infected with *L. donovani*. The lysates of *L. donovani*-infected macrophages were mixed with non-infected macrophage lysates in different ratios and coated on the 96-well plate so that each well contained the same amount of cell lysate from 3000 macrophages (infected plus non-infected) with amastigote numbers ranging from 100 to 6400 per well. As shown in Fig. 3b, the anti-1357 antiserum could detect down to approximately 200 amastigotes under these conditions and exhibited a *L. donovani* amastigote, dose-dependent increase in the signal. Similarly, signal was detected with human H1299 cell lysates mixed with *L. donovani* infected macrophages down to ~ 200 amastigotes (Fig. 3c). In contrast, although relatively strong signals were detected for the antiserum to *Leishmania* aldolase (36126), a significant background was also observed for this anti-36,126 serum (Fig. 3b and c), likely due to the high sequence identity for the *Leishmania* and human aldolase proteins. Taken together, out of the eight antisera, only the anti-1357 serum to the 40S ribosomal protein S12 gave applicable results and could detect approximately 200 amastigotes per 3000 macrophage lysates per well.

The weak-reacting signals for antigens 36,676, 3582 and 545 could have been due either to poor binding of these *Leishmania* proteins to the plate or poor recognition of these non-denatured or partially denatured proteins. All the antisera except 36,676 and 3582 showed good increases in signal intensity as the number of *L. donovani* promastigotes increased in the lysates, at least up to 100,000 promastigotes (Fig. 3a). However, only 36,126 antiserum produced a signal using 1 million *L. donovani* cells (see below).

It is noteworthy that a *Leishmania* sandwich ELISA developed by Ferrua et al. could detect circular *Leishmania* antigen in sera of *L. infantum* infected visceral leishmaniasis patients with sensitivity like that shown in Fig. 3 [18]. Since the maximum binding capacity of a 96-well ELISA plate well is about 1 μg protein, it is necessary to dilute the *Leishmania* lysate to a concentration of 20 μg/ ml (1 μg/50 μl/ well) in coating buffer. If the protein concentration is higher than 1 μg/well, then all the binding sites will be saturated and other proteins could compete with the target antigen for binding sites. This could result in loss of signal, consistent with our observation (Fig. 3a) at lysates from one million *L. donovani* promastigotes containing about 4 μg protein which could compete with target antigen binding [18].

### Sandwich ELISA for *Leishmania* 40S ribosomal protein S12 antigen

Since the binding capacity is limited in the direct ELISA, it could be difficult to detect the antigen if present at low levels or if they fail to adhere to the plastic. In this case, a sandwich (or capture) ELISA may be more sensitive since more of the antigen can be immobilized on the plate.

**Fig. 4:**
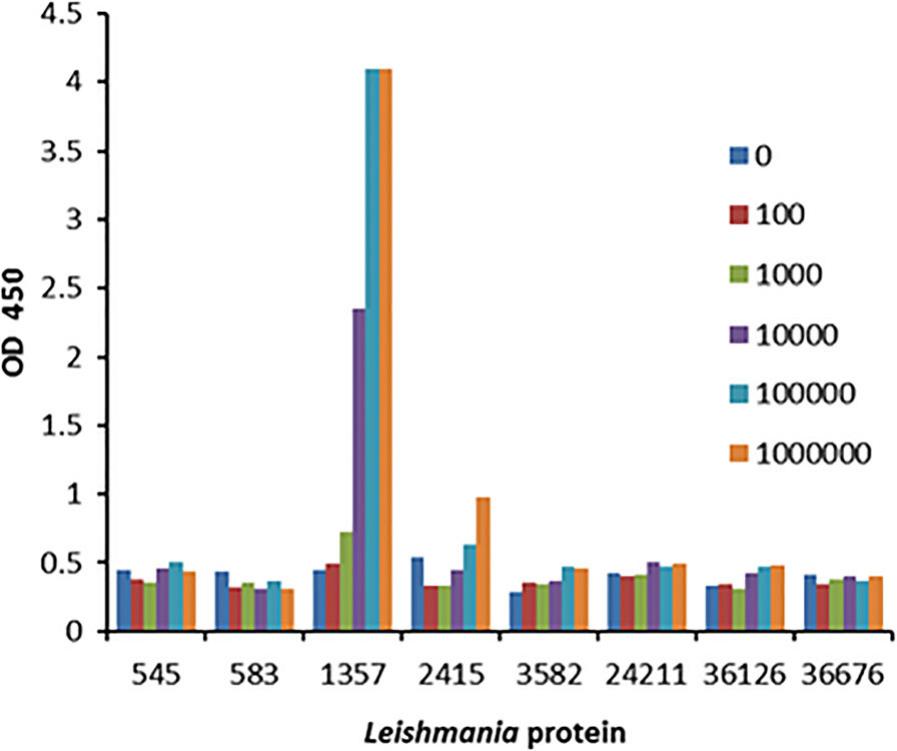
*Leishmania* 40S ribosomal protein S12 antigen (1357) sandwich ELISA had the highest sensitivity and strongest signal among the sandwich ELISAs prepared from the eight rabbit antisera. Unlabeled capture IgG and biotinylated detection IgG were prepared from each of the rabbit sera and tested in sandwich ELISAs. The capture IgG was used at a 1:2000 dilution. Whole cell lysates of 100 to 1 million *L. donovani* promastigotes were added to the wells of the 96-well ELISA plate. Captured antigen was detected with the homologous biotinylated IgG, Avidin-HRP, and TMB substrate, and absorbance measured at 450 nm

In this study the rabbit polyclonal antibodies were used as both the capture antibody and the detection antibody in the sandwich ELISA. Total IgG were purified from each of the rabbit antisera, unlabeled purified IgGs (1:2000 dilution) were coated on the ELISA plate as protein-capture antibodies, and one-half of the purified IgG was biotinylated as detection antibody. After blocking and washing, the *L. donovani* promastigote lysate ranging from 100 to 1 million cells was added into each well of the ELISA plate. The captured *Leishmania* antigen was detected with the biotin-labelled IgGs, avidin-HRP, and TMB substrate. As shown in the sandwich ELISA of Fig. 4, only the 40S ribosomal S12 antigen (1357) displayed a dose-dependent signal that became saturated beyond an input of 100,000 promastigotes.

**Fig. 5:**
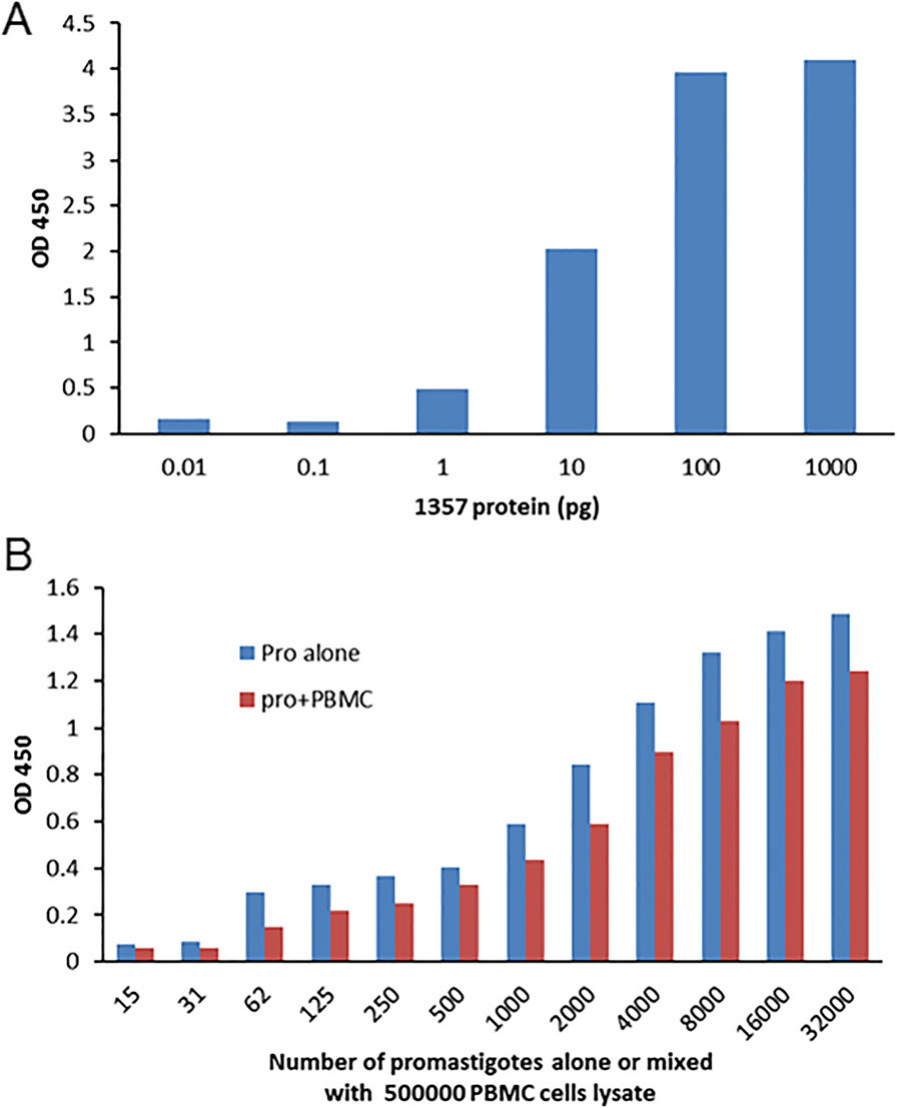
*Leishmania* 40S ribosomal protein S12 sandwich ELISA displayed high sensitivity and specificity. This ELISA assay can detect as low as 1 pg of purified recombinant 40S ribosomal protein S12 (**a**), the lysate of 60 *Leishmania donovani* parasites alone or mixed with 500,000 PBMCs prepared from healthy controls (**b**), which equals about 60 *Leishmania* amastigotes in 750 μl blood. Note: the detecting signal intensities were slightly lower in spiked PBMC lysate due to the matrix effect

The 40S ribosomal protein S12 (1357) sandwich ELISA was optimized by further dialysis to remove any free biotin conjugates, and various dilutions of capture antibody and detection antibody were tested to improve the signal to noise ratio. After optimization it was possible to detect as little as 1 pg of purified recombinant 40S ribosomal protein S12 (Fig. 5a) and as few as 60 *L. donovani* parasites either tested alone or mixed with 500,000 human PBMCs from healthy donors (Fig. 5b), which represents PBMCs from about 0.75 ml of blood.

### Analysis of clinical samples with the *Leishmania* 40S ribosomal protein S12 (1357) sandwich ELISA

**Fig. 6:**
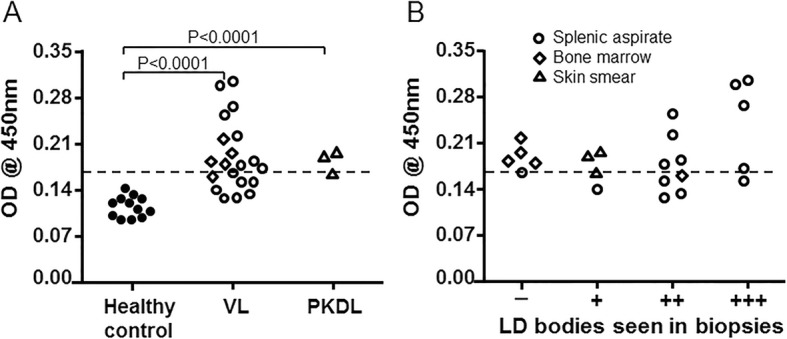
*Leishmania* 40S ribosomal protein S12 sandwich ELISA was able to detect the target antigen specifically in the clinical samples. **a** This novel ELISA detected *Leishmania* 40S ribosomal protein S12 antigen in lysates of PBMCs isolated from Visceral Leishmaniasis (VL, including 5 likely VL cases) and post-kala-azar dermal leishmaniasis (PKDL) patients before treatment. The A450 value differences are statistically significant (Student’s t-test); *P* < 0.0001 between the healthy controls and VL or PKDL patients. The dashed line is the standard cut-off value of the mean of healthy control +3SD (standard deviation). **b** This ELISA could detect the target antigen in lysates of PBMCs isolated from patients who were LD-body (Amastigote) negative in bone marrow or splenic biopsies, and the absorbance values for the target antigen were roughly correlated to the number of LD bodies seen in the spleen biopsies of VL patients. Open circles represent VL and likely VL cases examined by splenic aspirate; Open diamond, VL and likely VL case examined by bone marrow biopsy; Open triangle, PKDL patient confirmed by skin smear microscopy; Filled circle, healthy control

To determine whether this sandwich ELISA could detect the *Leishmania* 40S ribosomal protein S12 antigen from the clinical samples, PBMCs were isolated from 12 healthy controls, 14 VL patients, 3 post-kala-azar dermal leishmaniasis (PKDL) patients, and 5 likely VL cases before treatment at the Rejandra Memorial Research Institute of Medical Sciences (RMRIMS), Patna, Bihar, India. *Leishmania donovani* (LD) bodies (amastigotes) in the spleen or bone marrow biopsies were positive for the 14 confirmed VL patients, and negative for the 5 likely VL cases that were, however, positive on rK39 Rapid strip test and displayed clinical symptoms of VL including prolonged fever and enlarged spleen (Fig. 6b). All three PKDL cases were confirmed by skin smear microscopy for LD bodies and the 12 healthy controls were from the out-patient department and rK39 negative.

As shown in Fig. 6a, the A_450_ value differences were statistically significant (*P* < 0.0001) between these VL patients (median value, 0.191) and healthy controls (median value, 0.117) or between PKDL patients (median value, 0.185) and healthy controls. It is also noteworthy that while the ELISA values correlate roughly with the score of parasite levels from splenic biopsies (Fig. 6b), this ELISA was also capable of detecting the target 40S ribosomal protein S12 antigen in the PBMC samples of the 5 likely VL cases who were LD-body negative (Fig. 6b). If using the mean of the healthy controls +3SD (standard deviation) as the standard cut-off value (dashed line), 68% (15/22) of the rK39-positive cases (14 VL patients plus 5 likely VL cases) and 3 PKDL patients can be identified as *Leishmania* 40S ribosomal protein S12 antigen positive. Considering the sensitivity of this ELISA (Fig. 5b), this indicates that these VL patients could have approximately 15–80 amastigotes per ml of their blood.

## Discussion

An ELISA was developed to detect *Leishmania* proteins based on the assumption that abundant proteins could be more easily detected than proteins of low abundance in clinical samples. Antibodies raised against the 40S ribosomal protein S12 antigen (1357) outperformed all the other antisera tested and could detect the presence of *L. donovani* in PBMCs from VL patients. Although all the generated rabbit antisera could bind specifically to the corresponding *Leishmania* proteins in denatured form by Western blot analysis, antiserum to the 40S ribosomal S12 antigen was far superior to the other generated antibodies at recognizing their corresponding native *Leishmania* proteins in the ELISA. One explanation for this is that these *E. coli* expressed recombinant proteins were in a different, possibly denatured form that differs from that of the native *Leishmania* proteins. Moreover, we have recently found that antisera raised against some additional abundant *Leishmania* proteins (LdBPK_140910.1; LdBPK_250740.1; LdBPK_363750.1; LdBPK_321910.1 and LdBPK_180690.1) also failed to recognize the native *Leishmania* proteins despite working in Western blot analysis (data not shown). Therefore, future attempts should consider different methods for production and purification of recombinant proteins for generation of antibodies. Different adjuvants could also be considered to produce antibodies that recognize the native *Leishmania* proteins. Another relevant observation from this study is that we synthesized twelve peptide sequences (ten amino acid each) of the 40S ribosomal S12 antigen to generate anti-peptide monoclonal antibodies. However, possibly due to the native protein folding which may hide these epitopes, none of these anti-peptide monoclonal antibodies recognized the native 40S ribosomal protein S12 despite binding to the cognate peptides (See these peptides sequences in Methods).

The 40S ribosomal protein S12 antigen (1357) antiserum could be used to develop a sandwich ELISA that could detect as low as 1 pg purified recombinant 40S ribosomal protein S12 and approximately 60 *Leishmania donovani* parasites. Notably, this ELISA was able to detect the *Leishmania* 40S ribosomal protein S12 antigen in 68% PBMC samples of VL and PKDL patients including the likely VL cases that were LD-body negative in splenic or bone marrow biopsies, but rK39 positive with clinical symptoms. This ELISA also provided a quantitative estimation of the parasitemia for these positive cases. This suggests that this ELISA could be further developed to detect parasitemia for test of cure and relapse and could also be helpful to confirm VL diagnosis of equivocal cases.

While this project was in progress, three *Leishmania* antigen detection tests were reported including a triple *Leishmania* protein detection ELISA (DetectoGen Inc., USA), a *Leishmania* antigen ELISA (Kalon Biologicals Ltd., UK) and a *Leishmania* Antigen Detect™ ELISA (Infectious Disease Research Institute, USA) [20–23]. All these tests detect *Leishmania* antigens with slight differences in sensitivity from urine samples of VL patients, and all could potentially be used to monitor treatment progress. The *Leishmania* antigen ELISA and *Leishmania* Antigen Detect™ ELISA were developed using polyclonal antibodies raised against the whole *Leishmania donovani* cell lysate [23]. The DetectoGen ELISA was developed by raising polyclonal antibodies against three *Leishmania* proteins identified in VL patients’ urine samples [20]. Interestingly, like the 40S ribosomal protein S12 (16 kDa), all these three target proteins used in the DetectoGen Inc., capture ELISA (iron superoxide dismutase, 22 kDa; tryparedoxin, 17 kDa; and nuclear transport factor 2, 14 kDa) are also small and abundant *Leishmania* proteins. The 40S ribosomal protein S12 ELISA being reported in our study has similar sensitivity to the triple protein ELISA (DetectoGen), and is approximately two times more sensitive than the whole cell lysate (WCL) antigen ELISAs. The DetectoGen ELISA has a detection limit ranging from 4 to 100 pg/ml of the target antigens [20–22]. The Detect™ ELISA detection limit is 4 ng/ml, equivalent to about 100 *Leishmania* parasites per well [23]. We have yet to test whether the 40S ribosomal protein S12 antigen is present in VL patient urine but, if it is, it would be worth determining whether the 40S ribosomal protein S12-IgG complements those in the DetectoGen test, which is also based on anti-recombinant protein antibodies.

Compared with capture ELISAs based on individual antigens, the advantages of WCL capture ELISAs are (1) the ease of preparing the WCL antigens and (2) the greater range of *Leishmania* proteins (native or denatured) present in various clinical samples that could be recognized by the polyclonal antibodies, potentially resulting in a more sensitive test. In fact, the WCL ELISA may detect predominantly abundant *Leishmania* proteins that are also present in the urine samples. Based on its genome sequence, *Leishmania* has approximately 8000 potential proteins, and many of these proteins could significantly increase the likelihood of cross reactions to human proteins, which would increase the background signal. Therefore, it would be useful to compare all the available *Leishmania* antigen detection ELISAs side-by-side with various clinical samples including urine, blood and PBMCs. With this information, it may be possible to further improve the sensitivity and specificity for *Leishmania* antigen detection.

## Conclusions

Progress is described in the development of a sandwich ELISA for detecting *Leishmania* 40S ribosomal protein S12 in PBMCs of VL and PKDL patients. Though more clinical samples of VL patients and other parasitic diseases such as malaria and trypanosomiasis should be tested to verify its sensitivity and specificity, such a test would be useful for confirming VL diagnosis, relapse cases and monitoring treatment progress. With some refinement and/or in combination with other recently described *Leishmania* antigen-detecting reagents [20], more sensitive *Leishmania* antigen detection tests could be developed.

## Abbreviations

BSA: Bovine serum albumin
ELISA: Enzyme linked immunosorbent assay
IgG: Immunoglobulin G
IPTG: Isopropyl β-D-1-thiogalactopyranoside
PBMC: Peripheral blood mononuclear cell
PBS: Phosphate-buffered saline
PCR: Polymerase chain reaction
PKDL: Post-kala-azar dermal leishmaniasis
rK39 ICT: rK39 immunochromatographic test
SDS-PAGE: Sodium dodecyl sulfate polyacrylamide gel electrophoresis
VL: Visceral leishmaniasis
